# Fetal Hydrops Associated With 47,XXX: A Case Report and Literature Review

**DOI:** 10.7759/cureus.62552

**Published:** 2024-06-17

**Authors:** Shunya Sugai, Kazufumi Haino, Masako Hayashi, Jun Nirei, Kosuke Yoshihara, Koji Nishijima

**Affiliations:** 1 Obstetrics and Gynecology, Niigata University Medical and Dental Hospital, Niigata, JPN; 2 Pediatrics, Niigata University Medical and Dental Hospital, Niigata, JPN

**Keywords:** triple x syndrome, hydrops fetalis, congenital abnormality, chylothorax, chromosome disorder

## Abstract

This report aims to investigate the association between 47,XXX and fetal hydrops by examining a clinical case and performing a comprehensive review of the relevant literature. A 34-year-old Japanese woman, gravida 2, para 1, was diagnosed with fetal hydrops at 27 weeks’ gestation. Prenatal testing revealed a 47,XXX karyotype. Interventions included thoracocentesis and a thoracoamniotic shunt. A cesarean delivery was performed at 34 weeks and the female neonate initially had respiratory challenges. After 69 days in the neonatal intensive care unit, the infant was discharged in stable condition, and the 47,XXX karyotype was confirmed. This case may add evidence suggesting an association between 47,XXX and fetal hydrops. Chromosomal abnormalities are causes of fetal hydrops, but its association with 47,XXX remains unclear. Providing comprehensive information on this condition to couples is crucial, and considering the inclusion of fetal hydrops in the list of associated conditions might be advisable.

## Introduction

The occurrence of 47,XXX is not uncommon, with an estimated rate of 84/100,000 newborn girls affected [1]. A wide range of associated phenotypes of 47,XXX has been reported, including asymptomatic cases, non-specific physical characteristics, and developmental disorders [2]. While congenital disorders are uncommon, reported cases include cleft lip or palate, cardiac defects, and renal or genitourinary abnormalities [3].

Fetal hydrops is defined as the accumulation of fluid in two or more fetal serous cavities, which may include subcutaneous edema, ascites, and pleural or pericardial effusion [4]. Hydrops fetalis is classified into immune hydrops fetalis, which refers to cases caused by erythrocyte alloimmunization such as in pregnancies with blood type incompatibility, and non-immune hydrops fetalis, which encompasses all other cases [5]. Most fetal hydrops cases are non-immune fetal hydrops, with a reported prevalence ranging from 1 in 1,700 to 1 in 3,000 pregnancies [5]. The most common etiologies of non-immune fetal hydrops include cardiovascular causes, chromosomal anomalies, and hematologic abnormalities. Polyhydramnios and preterm birth frequently occur during pregnancy. The prognosis after delivery varies depending on the underlying etiology; however, if the infant is born preterm, the prognosis is generally poor [5,6].

There is limited information available regarding the effects of 47,XXX on the fetal period. In this report, we present a case of fetal hydrops that was diagnosed prenatally with 47,XXX and review the existing literature on fetal hydrops associated with 47,XXX.

## Case presentation

The patient was a 34-year-old Japanese woman who was gravida 2, para 1. The previous pregnancy had been uneventful, but she had undergone a cesarean delivery at full term due to the arrest of labor. The child was currently healthy. She had no prior medical complications or significant family history. This pregnancy was conceived naturally. Tocolysis using ritodrine hydrochloride was initiated at 23 weeks’ gestation because of threatened preterm labor at the referral hospital. Her condition remained stable, but bilateral fetal pleural effusion developed at 27 weeks’ gestation, which resulted in her referral to our Obstetrics Department.

**Figure 1:**
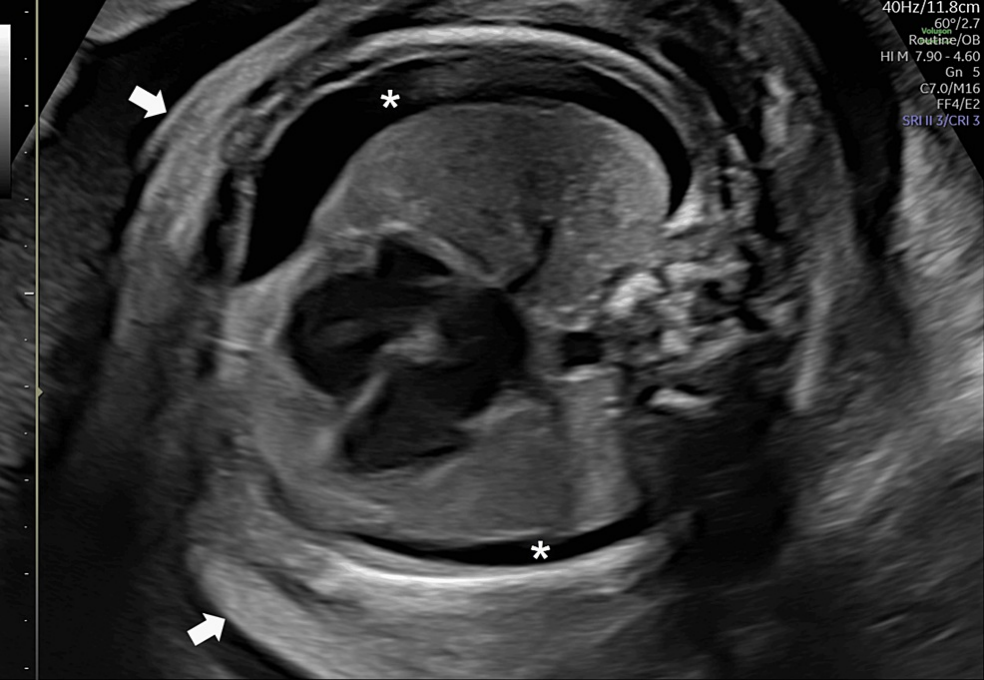
Fetal pleural effusion and subcutaneous edema at 28 weeks’ gestation (transverse section). Asterisks indicate pleural effusion, and arrows indicate subcutaneous edema.

We conducted an ultrasound examination on the day she was admitted to our department and found bilateral pleural effusion, ascites, and subcutaneous edema, confirming the diagnosis of fetal hydrops (Figure 1). Fetal growth was normal in size (1.0 standard deviation in estimated weight), and the amniotic fluid index was 22 cm. Doppler examination in the umbilical artery and middle cerebral artery were within the normal range. No structural abnormalities were observed. The maternal blood group was A RhD-positive, and antibody screening yielded negative results. Congenital infections including parvovirus, cytomegalovirus, syphilis, and toxoplasmosis were ruled out. We conducted daily ultrasound examinations and confirmed the condition of the fetus.

**Figure 2:**
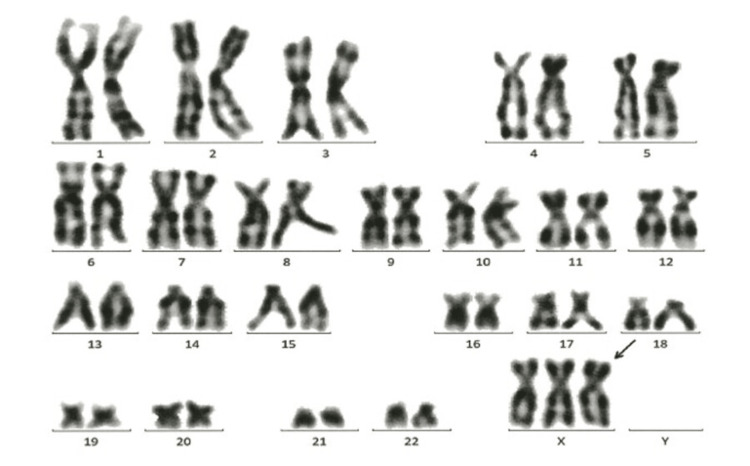
Chromosome test showing 47,XXX.

Fetal right pleural effusion continued to increase. Therefore, a unilateral thoracocentesis was performed at 30 weeks’ gestation, and approximately 20 mL of straw-colored pleural fluid was drained. This fluid was used to perform chromosome testing to search for the cause of fetal hydrops, and the testing showed 47,XXX karyotypes in all cells (Figure 2). After consultation with obstetricians and pediatricians, including a geneticist, we decided not to perform microarray testing and to perform G-banding only because no multiple complicating malformations were found. We offered the couple genetic counseling and provided comprehensive information regarding 47,XXX. The information included details about the frequency, potential malformations, developmental aspects, and fertility.

The fetal pleural effusion continued to increase, especially on the right side, but the position of the heart remained unchanged. Fetal Doppler studies showed normal findings. Fetal heart rate monitoring appeared to be reassuring. A unilateral fetal thoracoamniotic shunt was performed at 32 weeks’ gestation. Under ultrasound guidance, we inserted a 16-gauge puncture needle with a trocar into the fetal right thoracic cavity and placed a 4.5-Fr double-basket catheter (Hakko Co., Nagano, Japan). Postoperatively, the right pleural effusion had nearly disappeared, and expansion of the right lung was observed. Although the procedure was initially successful, the tube dislodged into the amniotic fluid two days later. Excessive amniotic fluid was removed three times at one-week intervals beginning at 31 weeks’ gestation. Approximately 1,000 mL of amniotic fluid was removed by aspiration with a syringe pump. There were no problems during and after the procedure. A cesarean delivery at 34 weeks’ gestation was conducted following a guideline [5]. A female neonate was delivered in a cephalic presentation, weighing 4,085 g, with a length of 47 cm. She had Apgar scores of 1 at one minute and 4 at five minutes and exhibited marked generalized subcutaneous edema. Neonatal blood gas analysis showed a pH of 7.28. The mother recovered and she was discharged seven days after the delivery.

The neonate was intubated after delivery, started on respiratory support, and admitted to the neonatal intensive care unit. She was carefully examined and had a normal morphology, except for pleural effusion, ascites, and subcutaneous edema. Oxygenation improved after the removal of the right pleural effusion. Pulmonary hypertension due to pulmonary hypoplasia was noted, necessitating the use of nitric oxide until day three of life. Additionally, she had a hemoglobin concentration of 12.0 g/dL, and red blood cell transfusion was performed. Furthermore, coagulation disorders and low albumin concentrations were observed, requiring the administration of fresh frozen plasma and albumin supplementation. Steroids and catecholamines were administered for circulatory failure. Following this administration, diuretics were provided, which resulted in improvement of the pleural effusion, ascites, and subcutaneous edema. Her overall condition gradually stabilized, and she was extubated on the 13th day post-birth. Chromosome G-banding was performed using peripheral lymphocytes, and it showed a 47,XXX karyotype consistent with the prenatal diagnosis. After a 69-day stay in the neonatal intensive care unit, she was discharged home in good condition. She showed normal growth and development at 10 months of age.

## Discussion

In this case, non-immune fetal hydrops developed, and the prenatal diagnosis showed that the fetus had a 47,XXX karyotype. Key mechanisms leading to non-immune hydrops fetalis include increased heart pressure, blood flow obstruction, inadequate ventricular filling, hepatic venous congestion, increased capillary permeability, anemia, lymphatic vessel dysplasia, and reduced osmotic pressure. The exact cause depends on the underlying disorder and often remains unclear [5,6].

We conducted a comprehensive review of articles documenting the co-occurrence of 47,XXX and fetal hydrops. We identified relevant articles in PubMed using the following search string: “triple X syndrome”[Supplementary Concept] OR “triple X syndrome”[All Fields] OR ((“trisomy”[All Fields] OR “trisomy”[MeSH Terms] OR “trisomy”[All Fields] OR “trisomies”[All Fields]) AND “X”[All Fields]) OR “47XXX”[All Fields].” All included articles were peer-reviewed and published in English from inception to August 23, 2023, and we also included relevant articles identified by manual searching. We included cases of mosaic 47,XXX/46,XX karyotypes, but excluded other mosaic karyotypes.

The results of the review are shown in Table 1. In our review, we identified three cases of fetal hydrops associated with 47,XXX [7-9]. In cases 2 and 3, chromosome testing was conducted owing to an advanced maternal age, and a 47,XXX karyotype was found. Subsequently, the findings of fetal hydrops followed the disclosure of the chromosome test results. In case 1 and our case, chromosomal analysis was performed to investigate fetal hydrops, leading to the identification of a 47,XXX karyotype. Case 3 presented as a mosaic with a 46,XX karyotype. Only case 1 showed multiple congenital disorders, none of which appeared to be directly related to fetal hydrops. Regarding pregnancy outcomes, case 1 resulted in stillbirth, while the other patients underwent cesarean delivery at 34-35 weeks’ gestation, and favorable progress was observed.

**Table 1:** Summary of a case of 47,XXX with fetal hydrops. CD: cesarean delivery; VD: vaginal delivery

Author, year	Maternal age	Weeks of fetal hydrops observed	Karyotype	Prenatal diagnosis	Materials	Reason	Weeks of delivery	Delivery mode	Congenital disorders	Perinatal outcome
Case 1: Spear, et al. 1988 [8]	31	28	47,XXX	Yes	Amniotic fluid	Scrutiny of fetal hydrops	29	VD	Gonadal dysgenesis, urinary tract malformation	Stillbirth
Case 2: Cardoso et al., 2001 [7]	42	-	47,XXX	Yes	Amniotic fluid	Advanced maternal age	35	CD	None	-
Case 3: Cremonini et al., 2014 [9]	41	34	47,XXX/46,XX	Yes	Chorionic villi	Advanced maternal age	34	CD	None	Healthy
Case 4: Our case	34	27	47,XXX	Yes	Pleural fluid	Scrutiny of fetal hydrops	34	CD	None	Healthy

The findings in our case and previous cases suggest that 47,XXX with fetal hydrops has a favorable prognosis when congenital disorders are absent. This possibility appears consistent with a previous report, which showed that 24 of 28 children diagnosed with non-immune edema had a normal neurological status and good long-term prognosis [10]. Generally, preterm delivery before 34 weeks’ gestation is recognized as an adverse prognostic factor in cases of fetal hydrops [5]. Therefore, delivery after 34 weeks’ gestation appears to be reasonable. If the fetus shows overall well-being, delivery at 37 weeks’ gestation or later may be considered, but physicians should remain vigilant for Mirror syndrome [5].

Chromosomal abnormalities are a common cause of fetal hydrops, accounting for 7%-16% of cases [5]. Among them, 21 trisomy and 45,X trisomy are well known, and 13/18 trisomy, 45,X mosaicism, 13 deletion, and 22q deletion have been reported [11-13]. The reports of fetal hydrops associated with 47,XXX are limited to the case reports that we reviewed. While these reports may strengthen the potential association between these two conditions, the etiology of this association remains unclear. One plausible hypothesis is the 45,X mosaicism proposed by Cardoso et al. [7]. The relationship between 47,XXX and fetal hydrops may be coincidental rather than inherent. The prevalence of 47,XXX is estimated to be 1 in 1,000 and that of fetal hydrops to be 1 in 3,000 [5,14]. Therefore, the coexistence of both conditions as a chance occurrence is plausible. The scarcity of reported cases may reflect publication bias.

With the increase in prenatal testing due to the rise in pregnancies among older gravidas, more cases of 47,XXX are expected to be detected early in pregnancy. Additionally, a report suggests that 47,XXX is associated with advanced maternal age [15]. When 47,XXX is identified, providing the affected couple with appropriate information is important. Including fetal hydrops in the list of congenital disorders associated with 47,XXX may be advisable.

## Conclusions

This case suggests a possible association between 47,XXX and fetal hydrops, highlighting the need for comprehensive information for affected couples and considering fetal hydrops as a related condition.
